# Crystal structure of 1-meth­oxy­pyrene

**DOI:** 10.1107/S2056989015003783

**Published:** 2015-02-28

**Authors:** Eric G. Morales-Espinoza, Ernesto Rivera, Reyna Reyes-Martínez, Simón Hernández-Ortega, David Morales-Morales

**Affiliations:** a Instituto de Investigaciones en Materiales, Universidad Nacional Autónoma de México, Circuito exterior, Ciudad Universitaria, México, D.F., 04510, Mexico; b Instituto de Química, Universidad Nacional Autónoma de México, Circuito Exterior, Ciudad Universitaria, México, D.F., 04510, Mexico

**Keywords:** crystal structure, pyrene, organic photovoltaics, π–π inter­actions, C—H⋯π inter­actions

## Abstract

The title compound, C_17_H_12_O, crystallized with three independent mol­ecules (*A*, *B* and *C*) in the asymmetric unit. In the crystal, the three independent mol­ecules are linked by π–π inter­actions [centroid–centroid distances = 3.551 (3)–3.977 (2) Å], which lead to the formation of trimers. Between the trimers there are a number of C—H⋯π inter­actions generating a laminar arrangement parallel to (010). The meth­oxy­methyl group in mol­ecule *A* is disordered over two sets of sites, with an occupancy ratio of 0.56 (9):0.44 (9).

## Related literature   

For information concerning π-conjugate systems, see: Dössel *et al.* (2012 ▸); Kim *et al.* (2008 ▸). For the synthesis of the title compound, see: Almeida *et al.* (2009 ▸). For details of the structures of pyrene and pyrene derivatives, see: Camerman & Trotter (1965 ▸); Gruber *et al.* (2006 ▸, 2010 ▸).
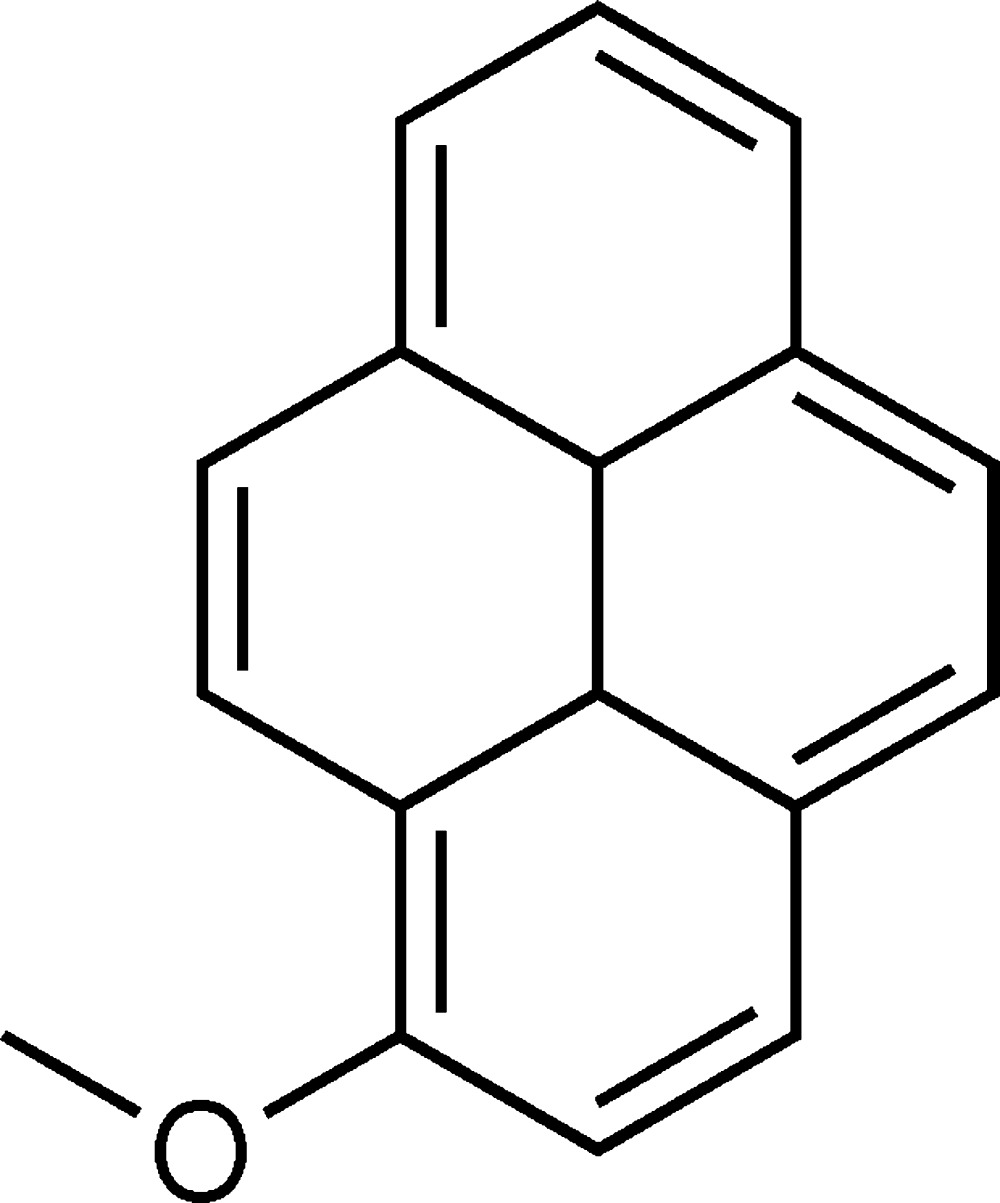


## Experimental   

### Crystal data  

C_17_H_12_O
*M* *_r_* = 232.27
Orthorhombic, 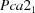

*a* = 16.4163 (15) Å
*b* = 15.8838 (15) Å
*c* = 13.5669 (13) Å
*V* = 3537.6 (6) Å^3^
*Z* = 12
Mo *K*α radiation
μ = 0.08 mm^−1^
*T* = 298 K
0.38 × 0.35 × 0.23 mm

### Data collection  

Bruker APEXII CCD diffractometer
15159 measured reflections
5670 independent reflections
3664 reflections with *I* > 2σ(*I*)
*R*_int_ = 0.103

### Refinement  

*R*[*F* ^2^ > 2σ(*F* ^2^)] = 0.058
*wR*(*F* ^2^) = 0.170
*S* = 1.01
5670 reflections
500 parameters
22 restraints
H-atom parameters constrained
Δρ_max_ = 0.19 e Å^−3^
Δρ_min_ = −0.17 e Å^−3^

### 

Data collection: *APEX2* (Bruker, 2012 ▸); cell refinement: *SAINT* (Bruker, 2012 ▸); data reduction: *SAINT*; program(s) used to solve structure: *SHELXS97* (Sheldrick, 2008 ▸); program(s) used to refine structure: *SHELXL2013* (Sheldrick, 2015 ▸); molecular graphics: *DIAMOND* (Brandenburg, 2006 ▸); software used to prepare material for publication: *SHELXL2013* and *DIAMOND*.

## Supplementary Material

Crystal structure: contains datablock(s) I. DOI: 10.1107/S2056989015003783/su5089sup1.cif

Structure factors: contains datablock(s) I. DOI: 10.1107/S2056989015003783/su5089Isup2.hkl

Supporting information file. DOI: 10.1107/S2056989015003783/su5089Isup3.cml

**Fig. 1.**
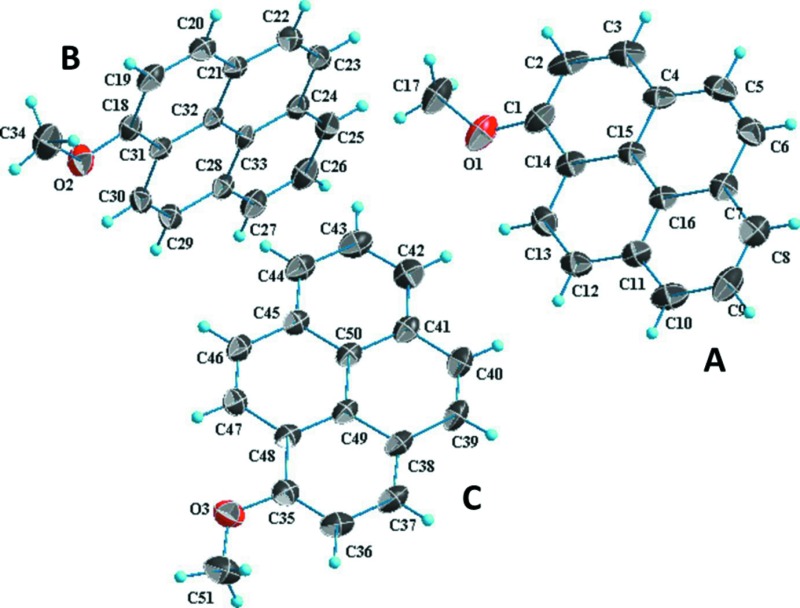
The molecular structure of the title compound, with atom labelling. Displacement ellipsoids are drawn at the 35% probability level. The minor component of the disordered methyl group of molecule A is not shown.

**Fig. 2.**
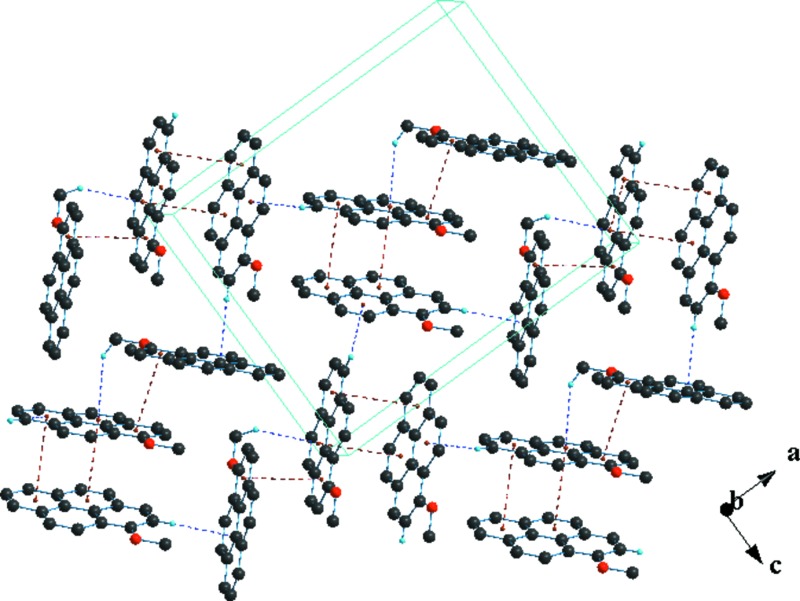
A view along the b axis of the crystal packing of the title compound, showing the laminar arrangement as a result of the π–π and C—H···π interactions (dashed lines; see Table 1 for details).

A . DOI: 10.1107/S2056989015003783/su5089fig1.tif
The mol­ecular structure of the title compound, with atom labelling. Displacement ellipsoids are drawn at the 35% probability level. The minor component of the disordered methyl group of mol­ecule *A* is not shown.

b . DOI: 10.1107/S2056989015003783/su5089fig2.tif
A view along the *b* axis of the crystal packing of the title compound, showing the laminar arrangement as a result of the π–π and C—H⋯π inter­actions (dashed lines; see Table 1 for details).

CCDC reference: 1050924

Additional supporting information:  crystallographic information; 3D view; checkCIF report

## Figures and Tables

**Table 1:** Hydrogen-bond geometry (, ) *Cg*1, *Cg*2, *Cg*3 and *Cg*4 are the centroids of the C38C41/C50/C49, C7C11/C16, C11C16, and C28C33 rings, respectively.

*D*H*A*	*D*H	H*A*	*D* *A*	*D*H*A*
C17H17*C* *Cg*1^i^	0.96	2.93	3.78(3)	148
C34H34*C* *Cg*2^i^	0.96	2.99	3.770(7)	140
C19H19*Cg*3^i^	0.93	2.99	3.733(6)	138
C44H44*Cg*4	0.93	2.64	3.529(6)	160

Symmetry code: (i) 
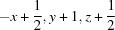
.

## supplementary crystallographic information

### S1. Introduction

π-conjugated aromatic compounds are promising materials for use in opto-electronic devices, particularly for organic photovoltaics (OPVs). These compounds exhibit both energy and charge transfer, and their absorption and emission wavelengths can be tuned (Dössel *et al.*, 2012). Pyrene derivatives have been extensively studied due to their excellent optical and electronic properties for example, excimer/monomer emission. Moreover, pyrene shows a long fluorescence lifetime which reaches 400 ns in cyclo­hexane solution (Kim *et al.*, 2008). Due to its susceptibility to aromatic substitution at the 1-, 3-, 6- and 8-positions, pyrene is often functionalized at these positions in order to improve its properties. In this context the title compound, 1-meth­oxy­pyrene, appears in the literature as an important inter­mediate in the synthesis of more elaborate compounds. Thus, in this context we report herein on the synthesis and crystal structure of the title compound.

### S2.1. Synthesis and crystallization

1-meth­oxy­pyrene was synthesized from 1-pyrenol which is synthesized from 1-pyrenecarboxaldehyde that is commercially available (Aldrich). Pyrenol (0.3 g, 1.37 mmol) was added to a solution of KOH (0.23 g, 4.12 mmol) dissolved in DMSO (7 mL). To this solution, methyl iodide (0.25 g, 1.78 mmol) was added and the resulting reaction mixture was stirred for 1 h at room temperature to produce the desired product (Almeida *et al.*, 2009). Yellow crystals of the title compound were obtained by recrystallization from CHCl_3_. ^1^H NMR (300 MHz, CDCl_3_): d = 8.47 (*d*, ArH, 1H), 8.09 (*m*, ArH, 4H), 7.94 (*m*, ArH, 4H), 7.55 (*d*, ArH, 1H), 4.17 (*s*, CH_3_, 3H) ppm.

### S2.2. Refinement

Crystal data, data collection and structure refinement details are summarized in Table 1. The H atoms were included in calculated positions and treated as riding: C—H = 0.93 - 0.96 Å with U_iso_(H) = 1.5U_eq_(C) for the methyl H atoms and = 1.2U_eq_(C) for other H atoms. The meth­oxy methyl group (C17) in molecule A is disordered over two sites with an occupancy ratio of 0.56 (9):0.44 (9).

### S3. Results and discussion

The asymmetric unit of the title compound consist of three independent molecules (A, B and C), as shown in Fig. 1. The pyrene moiety shows bond lengths and angles similar to those observed for free pyrene (Camerman & Trotter, 1965) and other pyrene derivatives (Gruber *et al.*, 2006, 2010).

In the crystal, the three molecules are linked by π-π inter­actions to give a trimeric motif. The distances between centroids of the aromatic rings have values in the range of 3.551 (3) to 3.977 (2) Å. The most significant are those between molecules A and C [Cg1···Cg9^i^ = 3.755 (3) Å, where Cg1 and Cg9 are the centroids of rings C1—C4/C15/C14 and C35—C38/C49/C48, respectively; symmetry code: (i) x+1/2, -y+1, z] and molecules B and C [Cg6···Cg12^ii^ = 3.551 (3) Å, where Cg6 and Cg12 are the centroids of rings C21—C24/C33/C32 and C45—C50, respectively; symmetry code: (ii) -x+1/2, y, z-1/2]. Between the trimmers there are C—H···π inter­actions generating a laminar arrangement parallel to the *ac* plane (Table 1 and Fig. 2).

### Crystal data

**Table:**

C_17_H_12_O	*D*_x_ = 1.308 Mg m^−^^3^
*M**_r_* = 232.27	Mo *K*α radiation, λ = 0.71073 Å
Orthorhombic, *P**c**a*2_1_	Cell parameters from 8039 reflections
*a* = 16.4163 (15) Å	θ = 2.3–25.4°
*b* = 15.8838 (15) Å	µ = 0.08 mm^−^^1^
*c* = 13.5669 (13) Å	*T* = 298 K
*V* = 3537.6 (6) Å^3^	Prism, colourless
*Z* = 12	0.38 × 0.35 × 0.23 mm
*F*(000) = 1464	

### Data collection

**Table:**

Bruker APEXII CCD diffractometer	*R*_int_ = 0.103
φ and ω scans	θ_max_ = 25.4°, θ_min_ = 1.3°
15159 measured reflections	*h* = −19→16
5670 independent reflections	*k* = −19→17
3664 reflections with *I* > 2σ(*I*)	*l* = −16→13

### Refinement

**Table:**

Refinement on *F*^2^	Hydrogen site location: inferred from neighbouring sites
Least-squares matrix: full	H-atom parameters constrained
*R*[*F*^2^ > 2σ(*F*^2^)] = 0.058	*w* = 1/[σ^2^(*F*_o_^2^) + (0.0923*P*)^2^] where *P* = (*F*_o_^2^ + 2*F*_c_^2^)/3
*wR*(*F*^2^) = 0.170	(Δ/σ)_max_ < 0.001
*S* = 1.00	Δρ_max_ = 0.19 e Å^−^^3^
5670 reflections	Δρ_min_ = −0.17 e Å^−^^3^
500 parameters	Extinction correction: *SHELXL2013* (Sheldrick, 2015), Fc^*^=kFc[1+0.001xFc^2^λ^3^/sin(2θ)]^-1/4^
22 restraints	Extinction coefficient: 0.0054 (14)

### Special details

**Table:**

Geometry. All e.s.d.'s (except the e.s.d. in the dihedral angle between two l.s. planes) are estimated using the full covariance matrix. The cell e.s.d.'s are taken into account individually in the estimation of e.s.d.'s in distances, angles and torsion angles; correlations between e.s.d.'s in cell parameters are only used when they are defined by crystal symmetry. An approximate (isotropic) treatment of cell e.s.d.'s is used for estimating e.s.d.'s involving l.s. planes.

### Fractional atomic coordinates and isotropic or equivalent isotropic displacement parameters (Å^2^)

**Table:**

	*x*	*y*	*z*	*U*_iso_*/*U*_eq_	Occ. (<1)
C1	0.2494 (4)	0.3042 (4)	0.8372 (4)	0.0827 (17)	
O1	0.2510 (3)	0.3889 (3)	0.8159 (4)	0.1116 (15)	
C17	0.305 (2)	0.433 (3)	0.749 (2)	0.104 (7)	0.56 (9)
H17A	0.2922	0.4922	0.7495	0.155*	0.56 (9)
H17B	0.2979	0.4116	0.6832	0.155*	0.56 (9)
H17C	0.3604	0.4252	0.7691	0.155*	0.56 (9)
C17A	0.316 (2)	0.400 (4)	0.743 (3)	0.112 (8)	0.44 (9)
H17D	0.3209	0.4580	0.7256	0.168*	0.44 (9)
H17E	0.3035	0.3675	0.6847	0.168*	0.44 (9)
H17F	0.3669	0.3802	0.7699	0.168*	0.44 (9)
C2	0.3000 (4)	0.2432 (6)	0.7968 (5)	0.101 (2)	
H2	0.3398	0.2589	0.7516	0.121*	
C3	0.2923 (4)	0.1605 (5)	0.8224 (5)	0.096 (2)	
H3	0.3259	0.1206	0.7930	0.115*	
C4	0.2357 (3)	0.1354 (4)	0.8909 (4)	0.0745 (15)	
C5	0.2263 (4)	0.0496 (4)	0.9180 (6)	0.093 (2)	
H5	0.2584	0.0087	0.8880	0.111*	
C6	0.1715 (4)	0.0267 (4)	0.9865 (5)	0.0872 (18)	
H6	0.1670	−0.0299	1.0032	0.105*	
C7	0.1209 (3)	0.0855 (4)	1.0339 (4)	0.0716 (15)	
C8	0.0652 (4)	0.0625 (4)	1.1054 (5)	0.0907 (19)	
H8	0.0611	0.0062	1.1237	0.109*	
C9	0.0168 (4)	0.1201 (5)	1.1492 (5)	0.102 (2)	
H9	−0.0197	0.1030	1.1977	0.123*	
C10	0.0206 (3)	0.2037 (5)	1.1234 (4)	0.0937 (19)	
H10	−0.0133	0.2425	1.1543	0.112*	
C11	0.0753 (3)	0.2306 (4)	1.0504 (4)	0.0681 (14)	
C12	0.0818 (4)	0.3160 (4)	1.0210 (4)	0.0802 (16)	
H12	0.0473	0.3557	1.0493	0.096*	
C13	0.1371 (4)	0.3414 (4)	0.9526 (4)	0.0759 (16)	
H13	0.1401	0.3978	0.9346	0.091*	
C14	0.1909 (3)	0.2813 (4)	0.9081 (4)	0.0689 (14)	
C15	0.1841 (3)	0.1954 (3)	0.9347 (4)	0.0603 (13)	
C16	0.1270 (3)	0.1717 (3)	1.0065 (3)	0.0611 (13)	
O2	0.3921 (2)	0.9290 (2)	0.8083 (3)	0.0832 (11)	
C18	0.4051 (3)	0.8500 (3)	0.7719 (4)	0.0615 (13)	
C19	0.4681 (3)	0.7987 (4)	0.8002 (4)	0.0679 (15)	
H19	0.5064	0.8178	0.8456	0.081*	
C20	0.4747 (3)	0.7192 (3)	0.7615 (4)	0.0658 (14)	
H20	0.5178	0.6853	0.7817	0.079*	
C21	0.4204 (3)	0.6878 (3)	0.6945 (3)	0.0544 (11)	
C22	0.4248 (3)	0.6049 (3)	0.6535 (4)	0.0657 (13)	
H22	0.4659	0.5688	0.6743	0.079*	
C23	0.3714 (3)	0.5778 (3)	0.5861 (4)	0.0671 (14)	
H23	0.3763	0.5234	0.5615	0.080*	
C24	0.3072 (3)	0.6302 (3)	0.5510 (4)	0.0567 (12)	
C25	0.2525 (4)	0.6041 (3)	0.4791 (4)	0.0705 (14)	
H25	0.2570	0.5504	0.4523	0.085*	
C26	0.1920 (4)	0.6570 (4)	0.4474 (4)	0.0802 (17)	
H26	0.1559	0.6387	0.3991	0.096*	
C27	0.1837 (3)	0.7364 (4)	0.4857 (4)	0.0717 (14)	
H27	0.1424	0.7714	0.4628	0.086*	
C28	0.2367 (3)	0.7654 (3)	0.5585 (3)	0.0548 (11)	
C29	0.2295 (3)	0.8470 (3)	0.6006 (4)	0.0625 (13)	
H29	0.1879	0.8823	0.5793	0.075*	
C30	0.2814 (3)	0.8747 (3)	0.6706 (4)	0.0612 (12)	
H30	0.2738	0.9276	0.6985	0.073*	
C31	0.3481 (3)	0.8234 (3)	0.7022 (3)	0.0493 (11)	
C32	0.3559 (3)	0.7414 (3)	0.6626 (3)	0.0468 (10)	
C33	0.3002 (3)	0.7124 (3)	0.5909 (3)	0.0469 (11)	
C34	0.4481 (4)	0.9611 (4)	0.8794 (5)	0.104 (2)	
H34A	0.4307	1.0158	0.9006	0.156*	
H34B	0.5013	0.9651	0.8504	0.156*	
H34C	0.4501	0.9238	0.9350	0.156*	
O3	−0.0805 (3)	0.8861 (3)	1.0803 (3)	0.0949 (13)	
C35	−0.0829 (3)	0.8032 (3)	1.0554 (4)	0.0718 (15)	
C36	−0.1395 (3)	0.7484 (4)	1.0944 (5)	0.0843 (17)	
H36	−0.1787	0.7674	1.1387	0.101*	
C37	−0.1370 (3)	0.6642 (4)	1.0665 (5)	0.0856 (18)	
H37	−0.1742	0.6270	1.0944	0.103*	
C38	−0.0814 (3)	0.6333 (4)	0.9990 (4)	0.0684 (14)	
C39	−0.0762 (3)	0.5470 (4)	0.9699 (5)	0.0816 (17)	
H39	−0.1114	0.5083	0.9986	0.098*	
C40	−0.0228 (4)	0.5201 (4)	0.9033 (5)	0.0830 (17)	
H40	−0.0220	0.4634	0.8863	0.100*	
C41	0.0339 (3)	0.5768 (4)	0.8569 (4)	0.0691 (15)	
C42	0.0902 (4)	0.5515 (4)	0.7864 (4)	0.0825 (16)	
H42	0.0914	0.4958	0.7655	0.099*	
C43	0.1446 (4)	0.6093 (5)	0.7471 (5)	0.0878 (18)	
H43	0.1813	0.5920	0.6990	0.105*	
C44	0.1451 (3)	0.6915 (4)	0.7781 (4)	0.0781 (16)	
H44	0.1829	0.7289	0.7517	0.094*	
C45	0.0903 (3)	0.7196 (3)	0.8481 (4)	0.0610 (13)	
C46	0.0894 (3)	0.8047 (3)	0.8818 (4)	0.0716 (15)	
H46	0.1278	0.8422	0.8573	0.086*	
C47	0.0350 (3)	0.8320 (3)	0.9476 (4)	0.0702 (15)	
H47	0.0362	0.8879	0.9678	0.084*	
C48	−0.0247 (3)	0.7770 (3)	0.9872 (4)	0.0607 (13)	
C49	−0.0249 (3)	0.6913 (3)	0.9580 (3)	0.0594 (13)	
C50	0.0328 (3)	0.6629 (3)	0.8874 (3)	0.0564 (12)	
C51	−0.1360 (4)	0.9177 (4)	1.1498 (6)	0.113 (2)	
H51A	−0.1230	0.9754	1.1642	0.170*	
H51B	−0.1327	0.8850	1.2092	0.170*	
H51C	−0.1902	0.9144	1.1236	0.170*	

### Atomic displacement parameters (Å^2^)

**Table:**

	*U*^11^	*U*^22^	*U*^33^	*U*^12^	*U*^13^	*U*^23^
C1	0.070 (4)	0.115 (5)	0.062 (4)	−0.023 (4)	−0.007 (3)	0.007 (3)
O1	0.103 (3)	0.135 (4)	0.097 (3)	−0.037 (3)	0.001 (3)	0.029 (3)
C17	0.095 (11)	0.128 (16)	0.088 (8)	−0.044 (11)	−0.007 (7)	0.025 (11)
C17A	0.115 (13)	0.13 (2)	0.094 (11)	−0.044 (14)	0.002 (7)	0.047 (14)
C2	0.055 (4)	0.185 (8)	0.063 (4)	−0.001 (4)	0.007 (3)	−0.011 (5)
C3	0.077 (4)	0.134 (6)	0.078 (4)	0.017 (4)	0.001 (4)	−0.013 (4)
C4	0.055 (3)	0.102 (5)	0.066 (3)	0.011 (3)	−0.009 (3)	−0.016 (3)
C5	0.077 (4)	0.098 (5)	0.103 (5)	0.030 (4)	−0.019 (4)	−0.024 (4)
C6	0.085 (4)	0.077 (4)	0.099 (5)	0.018 (3)	−0.020 (4)	0.004 (4)
C7	0.065 (3)	0.086 (4)	0.064 (3)	0.000 (3)	−0.023 (3)	0.008 (3)
C8	0.091 (5)	0.106 (5)	0.075 (4)	−0.006 (4)	−0.010 (4)	0.016 (4)
C9	0.098 (5)	0.133 (6)	0.076 (5)	−0.037 (5)	−0.001 (4)	0.027 (5)
C10	0.077 (4)	0.141 (6)	0.063 (4)	0.003 (4)	0.011 (3)	−0.014 (4)
C11	0.064 (3)	0.089 (4)	0.052 (3)	−0.004 (3)	−0.001 (3)	−0.008 (3)
C12	0.078 (4)	0.093 (5)	0.070 (4)	0.010 (3)	0.010 (3)	−0.019 (3)
C13	0.087 (4)	0.071 (4)	0.069 (4)	0.001 (3)	−0.013 (3)	−0.010 (3)
C14	0.058 (3)	0.097 (4)	0.051 (3)	−0.004 (3)	−0.006 (2)	−0.010 (3)
C15	0.052 (3)	0.076 (4)	0.052 (3)	0.006 (2)	−0.011 (2)	−0.005 (3)
C16	0.052 (3)	0.081 (4)	0.050 (3)	0.001 (3)	−0.012 (2)	−0.003 (3)
O2	0.084 (3)	0.078 (3)	0.087 (3)	−0.012 (2)	−0.023 (2)	−0.009 (2)
C18	0.063 (3)	0.067 (3)	0.054 (3)	−0.012 (3)	−0.003 (2)	0.002 (2)
C19	0.057 (3)	0.093 (4)	0.054 (3)	−0.017 (3)	−0.014 (2)	0.015 (3)
C20	0.052 (3)	0.080 (4)	0.065 (3)	0.004 (3)	−0.005 (3)	0.021 (3)
C21	0.049 (3)	0.065 (3)	0.049 (3)	0.001 (2)	0.005 (2)	0.020 (2)
C22	0.067 (3)	0.066 (3)	0.064 (3)	0.011 (3)	0.010 (3)	0.020 (3)
C23	0.079 (3)	0.055 (3)	0.067 (3)	0.002 (3)	0.021 (3)	0.006 (3)
C24	0.060 (3)	0.060 (3)	0.050 (3)	−0.012 (2)	0.015 (2)	0.008 (2)
C25	0.079 (4)	0.070 (4)	0.062 (3)	−0.022 (3)	0.011 (3)	−0.008 (3)
C26	0.078 (4)	0.100 (5)	0.063 (3)	−0.030 (4)	−0.012 (3)	−0.002 (3)
C27	0.066 (3)	0.083 (4)	0.066 (3)	−0.013 (3)	−0.013 (3)	0.012 (3)
C28	0.052 (3)	0.059 (3)	0.054 (3)	−0.008 (2)	−0.007 (2)	0.015 (2)
C29	0.060 (3)	0.053 (3)	0.074 (3)	0.002 (2)	−0.014 (3)	0.013 (2)
C30	0.067 (3)	0.046 (3)	0.070 (3)	0.000 (2)	−0.006 (3)	0.009 (2)
C31	0.048 (2)	0.056 (3)	0.044 (2)	−0.005 (2)	0.000 (2)	0.010 (2)
C32	0.049 (2)	0.049 (3)	0.042 (2)	−0.007 (2)	0.005 (2)	0.015 (2)
C33	0.053 (3)	0.048 (3)	0.039 (2)	−0.012 (2)	0.002 (2)	0.010 (2)
C34	0.108 (5)	0.106 (5)	0.098 (5)	−0.026 (4)	−0.025 (4)	−0.025 (4)
O3	0.095 (3)	0.089 (3)	0.101 (3)	0.021 (2)	0.016 (3)	0.015 (2)
C35	0.067 (3)	0.077 (4)	0.071 (4)	0.008 (3)	−0.005 (3)	0.024 (3)
C36	0.062 (3)	0.117 (5)	0.074 (4)	0.013 (3)	0.007 (3)	0.012 (4)
C37	0.056 (3)	0.116 (5)	0.085 (4)	−0.020 (3)	0.001 (3)	0.019 (4)
C38	0.048 (3)	0.092 (4)	0.065 (3)	−0.014 (3)	−0.004 (3)	0.015 (3)
C39	0.071 (4)	0.087 (4)	0.087 (4)	−0.033 (3)	−0.008 (3)	0.006 (3)
C40	0.078 (4)	0.085 (4)	0.086 (4)	−0.024 (3)	−0.009 (3)	−0.001 (3)
C41	0.059 (3)	0.086 (4)	0.062 (3)	−0.013 (3)	−0.014 (3)	0.009 (3)
C42	0.081 (4)	0.099 (4)	0.067 (4)	−0.007 (3)	−0.010 (3)	−0.008 (3)
C43	0.076 (4)	0.122 (6)	0.065 (4)	−0.003 (4)	0.006 (3)	0.011 (4)
C44	0.069 (4)	0.100 (5)	0.066 (4)	−0.006 (3)	0.003 (3)	0.025 (3)
C45	0.056 (3)	0.075 (4)	0.052 (3)	0.000 (3)	−0.004 (2)	0.026 (3)
C46	0.063 (3)	0.072 (4)	0.080 (4)	−0.004 (3)	−0.001 (3)	0.035 (3)
C47	0.071 (4)	0.062 (3)	0.078 (4)	0.005 (3)	−0.001 (3)	0.032 (3)
C48	0.051 (3)	0.074 (4)	0.056 (3)	0.004 (2)	−0.007 (2)	0.022 (3)
C49	0.046 (3)	0.076 (4)	0.056 (3)	−0.007 (2)	−0.010 (2)	0.021 (2)
C50	0.048 (3)	0.073 (3)	0.049 (3)	−0.003 (2)	−0.014 (2)	0.015 (2)
C51	0.109 (5)	0.120 (5)	0.111 (5)	0.039 (4)	0.014 (5)	0.000 (5)

### Geometric parameters (Å, º)

**Table:**

C1—O1	1.376 (7)	C24—C33	1.418 (6)
C1—C2	1.390 (9)	C25—C26	1.371 (8)
C1—C14	1.407 (8)	C25—H25	0.9300
O1—C17	1.452 (14)	C26—C27	1.372 (8)
O1—C17A	1.470 (18)	C26—H26	0.9300
C17—H17A	0.9600	C27—C28	1.394 (7)
C17—H17B	0.9600	C27—H27	0.9300
C17—H17C	0.9600	C28—C33	1.410 (6)
C17A—H17D	0.9600	C28—C29	1.421 (6)
C17A—H17E	0.9600	C29—C30	1.350 (7)
C17A—H17F	0.9600	C29—H29	0.9300
C2—C3	1.364 (9)	C30—C31	1.432 (6)
C2—H2	0.9300	C30—H30	0.9300
C3—C4	1.374 (8)	C31—C32	1.414 (6)
C3—H3	0.9300	C32—C33	1.412 (6)
C4—C15	1.406 (7)	C34—H34A	0.9600
C4—C5	1.421 (8)	C34—H34B	0.9600
C5—C6	1.343 (9)	C34—H34C	0.9600
C5—H5	0.9300	O3—C35	1.360 (6)
C6—C7	1.406 (8)	O3—C51	1.405 (8)
C6—H6	0.9300	C35—C36	1.379 (8)
C7—C8	1.382 (8)	C35—C48	1.394 (7)
C7—C16	1.423 (7)	C36—C37	1.391 (8)
C8—C9	1.351 (9)	C36—H36	0.9300
C8—H8	0.9300	C37—C38	1.382 (8)
C9—C10	1.375 (9)	C37—H37	0.9300
C9—H9	0.9300	C38—C49	1.421 (7)
C10—C11	1.403 (8)	C38—C39	1.428 (8)
C10—H10	0.9300	C39—C40	1.330 (8)
C11—C16	1.396 (7)	C39—H39	0.9300
C11—C12	1.418 (8)	C40—C41	1.439 (8)
C12—C13	1.360 (8)	C40—H40	0.9300
C12—H12	0.9300	C41—C42	1.390 (7)
C13—C14	1.434 (7)	C41—C50	1.429 (7)
C13—H13	0.9300	C42—C43	1.386 (8)
C14—C15	1.415 (7)	C42—H42	0.9300
C15—C16	1.403 (7)	C43—C44	1.373 (8)
O2—C18	1.365 (5)	C43—H43	0.9300
O2—C34	1.427 (6)	C44—C45	1.381 (7)
C18—C19	1.373 (7)	C44—H44	0.9300
C18—C31	1.395 (6)	C45—C50	1.409 (6)
C19—C20	1.372 (7)	C45—C46	1.428 (7)
C19—H19	0.9300	C46—C47	1.335 (8)
C20—C21	1.368 (7)	C46—H46	0.9300
C20—H20	0.9300	C47—C48	1.418 (7)
C21—C32	1.427 (6)	C47—H47	0.9300
C21—C22	1.431 (7)	C48—C49	1.418 (7)
C22—C23	1.338 (7)	C49—C50	1.421 (7)
C22—H22	0.9300	C51—H51A	0.9600
C23—C24	1.425 (7)	C51—H51B	0.9600
C23—H23	0.9300	C51—H51C	0.9600
C24—C25	1.389 (7)		
			
O1—C1—C2	126.0 (6)	C24—C25—H25	119.8
O1—C1—C14	114.2 (6)	C25—C26—C27	121.1 (5)
C2—C1—C14	119.8 (6)	C25—C26—H26	119.4
C1—O1—C17	128 (2)	C27—C26—H26	119.4
C1—O1—C17A	106 (3)	C26—C27—C28	120.7 (5)
O1—C17—H17A	109.5	C26—C27—H27	119.7
O1—C17—H17B	109.5	C28—C27—H27	119.7
H17A—C17—H17B	109.5	C27—C28—C33	119.0 (5)
O1—C17—H17C	109.5	C27—C28—C29	122.3 (5)
H17A—C17—H17C	109.5	C33—C28—C29	118.7 (4)
H17B—C17—H17C	109.5	C30—C29—C28	121.9 (4)
O1—C17A—H17D	109.5	C30—C29—H29	119.0
O1—C17A—H17E	109.5	C28—C29—H29	119.0
H17D—C17A—H17E	109.5	C29—C30—C31	120.5 (4)
O1—C17A—H17F	109.5	C29—C30—H30	119.8
H17D—C17A—H17F	109.5	C31—C30—H30	119.8
H17E—C17A—H17F	109.5	C18—C31—C32	118.5 (4)
C3—C2—C1	121.0 (6)	C18—C31—C30	122.8 (4)
C3—C2—H2	119.5	C32—C31—C30	118.6 (4)
C1—C2—H2	119.5	C33—C32—C31	120.3 (4)
C2—C3—C4	121.0 (6)	C33—C32—C21	119.6 (4)
C2—C3—H3	119.5	C31—C32—C21	120.1 (4)
C4—C3—H3	119.5	C28—C33—C32	119.9 (4)
C3—C4—C15	119.8 (6)	C28—C33—C24	119.4 (4)
C3—C4—C5	121.8 (6)	C32—C33—C24	120.8 (4)
C15—C4—C5	118.4 (6)	O2—C34—H34A	109.5
C6—C5—C4	120.8 (6)	O2—C34—H34B	109.5
C6—C5—H5	119.6	H34A—C34—H34B	109.5
C4—C5—H5	119.6	O2—C34—H34C	109.5
C5—C6—C7	122.2 (6)	H34A—C34—H34C	109.5
C5—C6—H6	118.9	H34B—C34—H34C	109.5
C7—C6—H6	118.9	C35—O3—C51	119.6 (5)
C8—C7—C6	122.4 (6)	O3—C35—C36	122.4 (6)
C8—C7—C16	119.0 (6)	O3—C35—C48	115.7 (5)
C6—C7—C16	118.6 (6)	C36—C35—C48	121.9 (6)
C9—C8—C7	121.3 (6)	C35—C36—C37	118.9 (6)
C9—C8—H8	119.3	C35—C36—H36	120.6
C7—C8—H8	119.3	C37—C36—H36	120.6
C8—C9—C10	121.0 (6)	C38—C37—C36	122.8 (5)
C8—C9—H9	119.5	C38—C37—H37	118.6
C10—C9—H9	119.5	C36—C37—H37	118.6
C9—C10—C11	120.2 (6)	C37—C38—C49	117.4 (5)
C9—C10—H10	119.9	C37—C38—C39	124.2 (5)
C11—C10—H10	119.9	C49—C38—C39	118.4 (5)
C16—C11—C10	119.1 (6)	C40—C39—C38	122.4 (5)
C16—C11—C12	118.3 (5)	C40—C39—H39	118.8
C10—C11—C12	122.5 (6)	C38—C39—H39	118.8
C13—C12—C11	121.7 (5)	C39—C40—C41	121.5 (6)
C13—C12—H12	119.1	C39—C40—H40	119.3
C11—C12—H12	119.1	C41—C40—H40	119.3
C12—C13—C14	120.0 (5)	C42—C41—C50	119.0 (5)
C12—C13—H13	120.0	C42—C41—C40	123.3 (6)
C14—C13—H13	120.0	C50—C41—C40	117.7 (5)
C1—C14—C15	118.5 (5)	C43—C42—C41	120.1 (6)
C1—C14—C13	122.4 (6)	C43—C42—H42	120.0
C15—C14—C13	119.1 (5)	C41—C42—H42	120.0
C16—C15—C4	120.9 (5)	C44—C43—C42	121.1 (6)
C16—C15—C14	119.2 (5)	C44—C43—H43	119.5
C4—C15—C14	119.8 (5)	C42—C43—H43	119.5
C11—C16—C15	121.5 (5)	C43—C44—C45	120.9 (5)
C11—C16—C7	119.4 (5)	C43—C44—H44	119.6
C15—C16—C7	119.1 (5)	C45—C44—H44	119.6
C18—O2—C34	118.1 (4)	C44—C45—C50	119.4 (5)
O2—C18—C19	124.2 (5)	C44—C45—C46	122.2 (5)
O2—C18—C31	114.8 (4)	C50—C45—C46	118.4 (5)
C19—C18—C31	121.0 (5)	C47—C46—C45	122.0 (5)
C20—C19—C18	119.9 (5)	C47—C46—H46	119.0
C20—C19—H19	120.0	C45—C46—H46	119.0
C18—C19—H19	120.0	C46—C47—C48	121.0 (5)
C21—C20—C19	122.6 (5)	C46—C47—H47	119.5
C21—C20—H20	118.7	C48—C47—H47	119.5
C19—C20—H20	118.7	C35—C48—C49	118.1 (5)
C20—C21—C32	117.9 (4)	C35—C48—C47	122.7 (5)
C20—C21—C22	124.1 (5)	C49—C48—C47	119.2 (5)
C32—C21—C22	118.0 (4)	C48—C49—C50	119.4 (4)
C23—C22—C21	121.9 (5)	C48—C49—C38	121.0 (5)
C23—C22—H22	119.1	C50—C49—C38	119.6 (5)
C21—C22—H22	119.1	C45—C50—C49	120.0 (5)
C22—C23—C24	121.7 (5)	C45—C50—C41	119.5 (5)
C22—C23—H23	119.2	C49—C50—C41	120.5 (4)
C24—C23—H23	119.2	O3—C51—H51A	109.5
C25—C24—C33	119.4 (5)	O3—C51—H51B	109.5
C25—C24—C23	122.5 (5)	H51A—C51—H51B	109.5
C33—C24—C23	118.1 (4)	O3—C51—H51C	109.5
C26—C25—C24	120.4 (5)	H51A—C51—H51C	109.5
C26—C25—H25	119.8	H51B—C51—H51C	109.5
			
C2—C1—O1—C17	−1 (2)	O2—C18—C31—C30	0.3 (6)
C14—C1—O1—C17	178.2 (19)	C19—C18—C31—C30	180.0 (4)
C2—C1—O1—C17A	0 (2)	C29—C30—C31—C18	177.9 (4)
C14—C1—O1—C17A	179 (2)	C29—C30—C31—C32	−3.4 (7)
O1—C1—C2—C3	−179.1 (6)	C18—C31—C32—C33	−179.2 (4)
C14—C1—C2—C3	2.1 (9)	C30—C31—C32—C33	2.0 (6)
C1—C2—C3—C4	−1.8 (10)	C18—C31—C32—C21	0.4 (6)
C2—C3—C4—C15	0.8 (9)	C30—C31—C32—C21	−178.3 (4)
C2—C3—C4—C5	179.5 (6)	C20—C21—C32—C33	177.9 (4)
C3—C4—C5—C6	178.8 (6)	C22—C21—C32—C33	−1.4 (6)
C15—C4—C5—C6	−2.5 (8)	C20—C21—C32—C31	−1.8 (6)
C4—C5—C6—C7	0.5 (9)	C22—C21—C32—C31	178.9 (4)
C5—C6—C7—C8	−179.1 (5)	C27—C28—C33—C32	178.2 (4)
C5—C6—C7—C16	1.3 (8)	C29—C28—C33—C32	−1.2 (6)
C6—C7—C8—C9	−179.5 (6)	C27—C28—C33—C24	−1.8 (6)
C16—C7—C8—C9	0.2 (8)	C29—C28—C33—C24	178.8 (4)
C7—C8—C9—C10	0.7 (10)	C31—C32—C33—C28	0.2 (6)
C8—C9—C10—C11	0.0 (9)	C21—C32—C33—C28	−179.4 (4)
C9—C10—C11—C16	−1.6 (8)	C31—C32—C33—C24	−179.8 (4)
C9—C10—C11—C12	−179.9 (6)	C21—C32—C33—C24	0.6 (6)
C16—C11—C12—C13	−0.6 (8)	C25—C24—C33—C28	1.4 (6)
C10—C11—C12—C13	177.7 (5)	C23—C24—C33—C28	−179.3 (4)
C11—C12—C13—C14	0.0 (8)	C25—C24—C33—C32	−178.6 (4)
O1—C1—C14—C15	179.7 (4)	C23—C24—C33—C32	0.7 (6)
C2—C1—C14—C15	−1.4 (8)	C51—O3—C35—C36	1.5 (8)
O1—C1—C14—C13	1.0 (7)	C51—O3—C35—C48	−178.9 (5)
C2—C1—C14—C13	179.8 (5)	O3—C35—C36—C37	−178.8 (5)
C12—C13—C14—C1	−179.8 (5)	C48—C35—C36—C37	1.5 (8)
C12—C13—C14—C15	1.5 (7)	C35—C36—C37—C38	−1.8 (9)
C3—C4—C15—C16	−178.6 (5)	C36—C37—C38—C49	0.4 (8)
C5—C4—C15—C16	2.7 (7)	C36—C37—C38—C39	178.9 (6)
C3—C4—C15—C14	−0.1 (7)	C37—C38—C39—C40	178.7 (5)
C5—C4—C15—C14	−178.9 (5)	C49—C38—C39—C40	−2.8 (8)
C1—C14—C15—C16	178.9 (4)	C38—C39—C40—C41	0.6 (8)
C13—C14—C15—C16	−2.3 (7)	C39—C40—C41—C42	−179.4 (5)
C1—C14—C15—C4	0.5 (7)	C39—C40—C41—C50	2.2 (8)
C13—C14—C15—C4	179.2 (4)	C50—C41—C42—C43	0.4 (7)
C10—C11—C16—C15	−178.6 (4)	C40—C41—C42—C43	−178.0 (5)
C12—C11—C16—C15	−0.2 (7)	C41—C42—C43—C44	1.3 (8)
C10—C11—C16—C7	2.4 (7)	C42—C43—C44—C45	−1.3 (9)
C12—C11—C16—C7	−179.2 (5)	C43—C44—C45—C50	−0.5 (8)
C4—C15—C16—C11	−179.9 (5)	C43—C44—C45—C46	179.9 (5)
C14—C15—C16—C11	1.7 (7)	C44—C45—C46—C47	178.3 (5)
C4—C15—C16—C7	−0.9 (7)	C50—C45—C46—C47	−1.3 (7)
C14—C15—C16—C7	−179.4 (4)	C45—C46—C47—C48	0.1 (8)
C8—C7—C16—C11	−1.8 (7)	O3—C35—C48—C49	−179.5 (4)
C6—C7—C16—C11	177.9 (5)	C36—C35—C48—C49	0.1 (7)
C8—C7—C16—C15	179.3 (4)	O3—C35—C48—C47	1.6 (7)
C6—C7—C16—C15	−1.1 (7)	C36—C35—C48—C47	−178.8 (5)
C34—O2—C18—C19	0.9 (7)	C46—C47—C48—C35	−179.5 (5)
C34—O2—C18—C31	−179.5 (4)	C46—C47—C48—C49	1.6 (7)
O2—C18—C19—C20	178.1 (5)	C35—C48—C49—C50	178.9 (4)
C31—C18—C19—C20	−1.6 (7)	C47—C48—C49—C50	−2.1 (6)
C18—C19—C20—C21	0.1 (8)	C35—C48—C49—C38	−1.6 (7)
C19—C20—C21—C32	1.5 (7)	C47—C48—C49—C38	177.3 (4)
C19—C20—C21—C22	−179.2 (5)	C37—C38—C49—C48	1.3 (7)
C20—C21—C22—C23	−178.2 (4)	C39—C38—C49—C48	−177.3 (4)
C32—C21—C22—C23	1.1 (7)	C37—C38—C49—C50	−179.2 (4)
C21—C22—C23—C24	0.2 (7)	C39—C38—C49—C50	2.2 (7)
C22—C23—C24—C25	178.2 (4)	C44—C45—C50—C49	−178.8 (4)
C22—C23—C24—C33	−1.1 (7)	C46—C45—C50—C49	0.8 (6)
C33—C24—C25—C26	−0.4 (7)	C44—C45—C50—C41	2.1 (6)
C23—C24—C25—C26	−179.7 (5)	C46—C45—C50—C41	−178.3 (4)
C24—C25—C26—C27	−0.1 (8)	C48—C49—C50—C45	0.9 (6)
C25—C26—C27—C28	−0.3 (8)	C38—C49—C50—C45	−178.5 (4)
C26—C27—C28—C33	1.3 (7)	C48—C49—C50—C41	179.9 (4)
C26—C27—C28—C29	−179.4 (5)	C38—C49—C50—C41	0.5 (6)
C27—C28—C29—C30	−179.5 (4)	C42—C41—C50—C45	−2.1 (7)
C33—C28—C29—C30	−0.2 (7)	C40—C41—C50—C45	176.4 (4)
C28—C29—C30—C31	2.5 (7)	C42—C41—C50—C49	178.9 (4)
O2—C18—C31—C32	−178.4 (4)	C40—C41—C50—C49	−2.6 (7)
C19—C18—C31—C32	1.2 (7)		

### Hydrogen-bond geometry (Å, º)

Cg1, Cg2, Cg3 and Cg4 are the centroids of the C38–C41/C50/C49, C7–C11/C16, C11–C16, and C28–C33 rings, respectively.

**Table:**

*D*—H···*A*	*D*—H	H···*A*	*D*···*A*	*D*—H···*A*
C17—H17*C*···*Cg*1^i^	0.96	2.93	3.78 (3)	148
C34—H34*C*···*Cg*2^i^	0.96	2.99	3.770 (7)	140
C19—H19···*Cg*3^i^	0.93	2.99	3.733 (6)	138
C44—H44···*Cg*4	0.93	2.64	3.529 (6)	160

Symmetry code: (i) −*x*+1/2, *y*+1, *z*+1/2.
