# Supplementary figures and images for: Crystal structure of 1-meth­oxy­pyrene
Source: Acta Crystallogr E Crystallogr Commun. 2015 Feb 28;71(Pt 3):o210–1. doi: 10.1107/S2056989015003783 (PMC4350705; doi:10.1107/S2056989015003783)

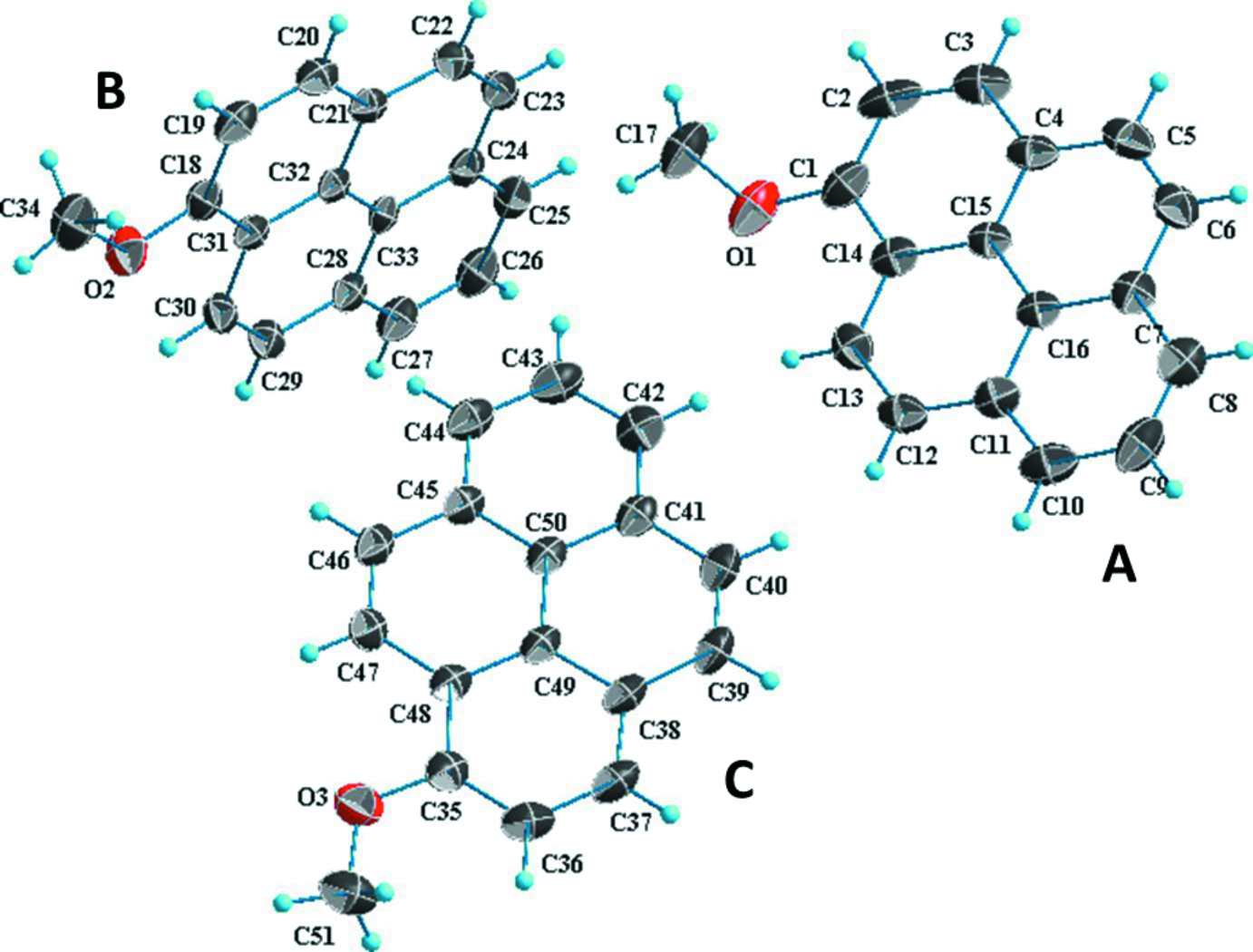

Supplement: Supplementary file 4 [file e-71-0o210-fig1.tif]

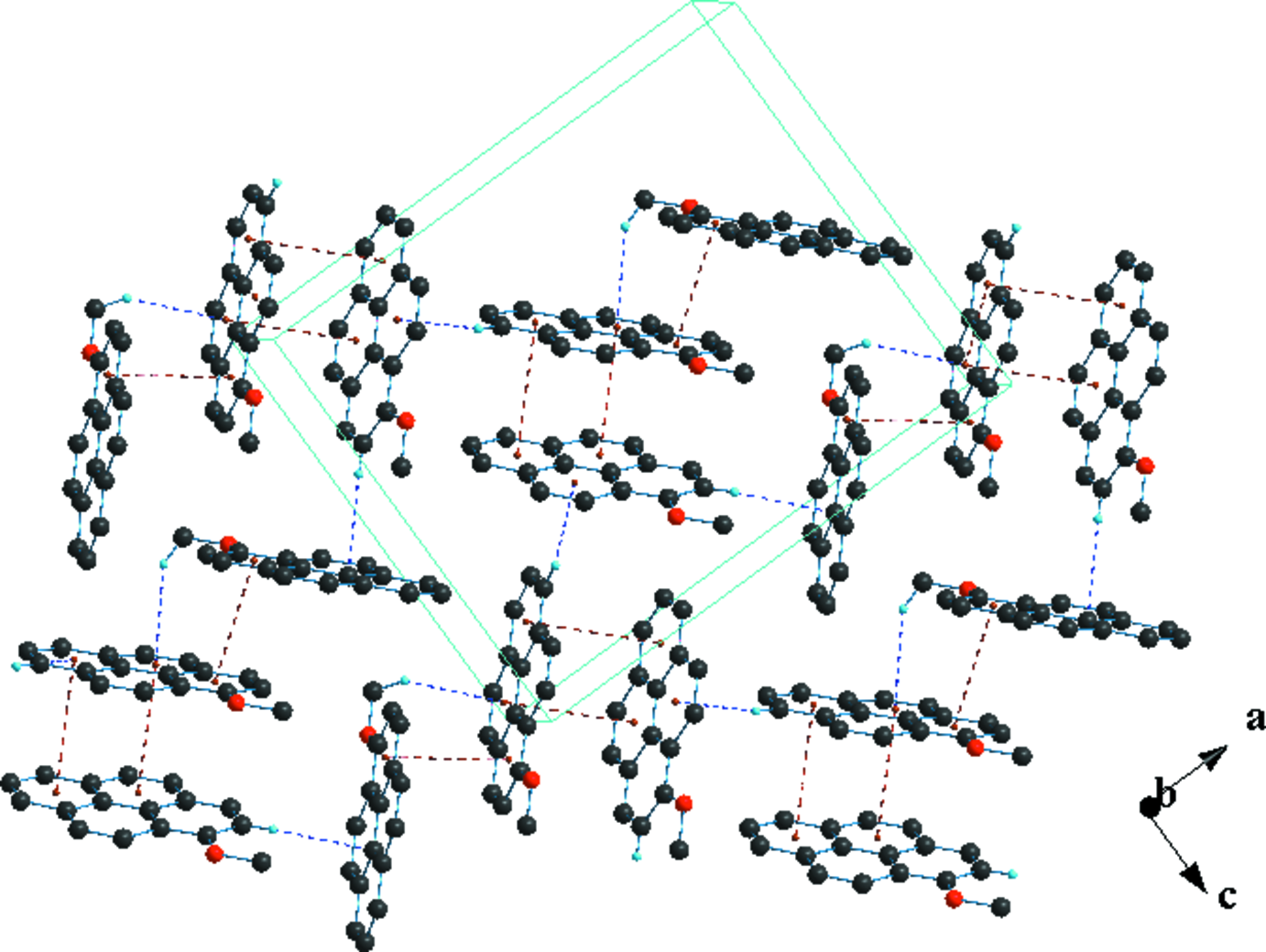

Supplement: Supplementary file 5 [file e-71-0o210-fig2.tif]
